# Administering the Union citizen in need: Between welfare state bureaucracy and migration control

**DOI:** 10.1177/0958928721999612

**Published:** 2021-04-12

**Authors:** Dion Kramer, Anita Heindlmaier

**Affiliations:** Vrije Universiteit Amsterdam – Transnational Legal Studies, Netherlands; Universität Salzburg – Salzburg Centre of European Union Studies/Department of Political Science, Austria

**Keywords:** administration, bureaucracy, EU citizenship, European Union, free movement, right of residence, social assistance, welfare state

## Abstract

How to determine whether mobile Union citizens have a right to social assistance? Research has shown how Western European Member States have made efforts to restrict Union citizens’ access to their welfare systems over the past decade, whereby lawful residence has increasingly become the linchpin for entitlement. Member States have responded strikingly differently, however, to the complex administrative puzzle of dealing with open borders, the ability to verify lawful residence and the right to social assistance over time. This article makes an analytical and empirical contribution to existing literature by asking how Member States adjust their welfare/migration administrations to fit the Union’s free movement regime and what implications this has for Union citizens. Based upon comparative case studies into the administration of social assistance rights in Germany, Austria and the Netherlands, the article develops a typology of three different models of administering Union citizens’ access to the welfare state: the *form*, *signal* and *delegation* models. Demonstrating how bureaucratic design impacts the stratification of social rights in the Member States in different ways, the article concludes that studying alternative administrative models offers important insights into the functioning of territorial welfare states in open border regimes.

## Introduction

The social rights enjoyed by immigrants depend on a country’s immigration policy and its welfare system. Compared to ‘third-country nationals’, mobile Union citizens have, as ‘privileged’ immigrants, a strong position in their welfare state of destination (see already Brubaker, 1989: 155–158; Sainsbury, 2012: 130). Indeed, the principle of equal treatment, connected to the status of Union citizenship, has rendered their discriminatory treatment in the field of social benefits by Member States (MS) increasingly difficult. Largely driven by controversial case law of the European Court of Justice (ECJ), more and more EU nationals have come to enjoy transnational social rights since the 1970s (Wollenschläger, 2011). But while Union citizenship, as introduced by the Treaty of Maastricht, might once have held the promise of a truly post-national social membership (Soysal, 1994), scholars have more recently observed the formation of a stratification of social rights for mobile Union citizens in practice (among others Bruzelius and Seeleib-Kaiser, 2017; Carmel and Paul, 2013). Especially during the past 10 years, against the background of increasing politicization of alleged ‘social tourism’, Western European MS have made efforts to restrict Union citizens’ access to their welfare states (Barbulescu and Favell, 2020; Mantu and Minderhoud, 2019; Roos, 2016).

A clear trend that emerges from this concerted move towards restrictive policies is that MS increasingly challenge the *legality* of the residence of Union citizens who resort to their welfare systems. As ‘the only gate [. . .] still under partial operational control of state administrations’ (Ferrera, 2014: 831), this is hardly surprising. After all, the right to reside in another MS is far from unconditional, as it requires economic activity or self-sufficiency after 3 months of residence (Shutes, 2016), and MS have increasingly discovered how to use these so-called ‘residence conditions’ against indigent Union citizens who apply for non-contributory social benefits (Kramer et al., 2018). In the process, they found an unexpected ally in the ECJ, which had earlier challenged MS’ attempts to restrict welfare provision on this ground, but now endorsed restrictive practices in cases such as *Dano* (2014) and *Commission v. UK* (2016) (Blauberger et al., 2018; O’Brien, 2016, 2017).

With *lawful residence* becoming the linchpin for welfare access, however, MS are confronted with an administrative challenge that goes to the very nexus between welfare state bureaucracy and migration control: how to combine the ability to verify lawful residence as a precondition for accessing welfare with the imperatives of open borders and free movement in the European Union? This article makes an analytical and empirical contribution to existing literature by (comparatively) studying the administrative nexus between the right to social assistance and the right of residence (for related research, see Bruzelius, 2019). It asks how – in response to the requirements of EU law and in light of the broader political context – MS (re)organize the administration of lawful residence in connection to Union citizens’ access to social assistance. It thereby contributes, first, to our understanding of how EU free movement law – ECJ jurisprudence in particular – challenges national welfare administrations and stimulates their adaptation, a topic largely ignored in research (see Blauberger and Schmidt, 2017). Second, by focusing on the specific effects and consequences of administrative adjustment and bureaucratic reorganization, the article sheds further light on forms of social stratification, by which we mean differentiation in the enjoyment of social rights by residents within a welfare state, that are taking shape in different MS.

In order to do so, the article starts by explaining the legal ambiguity and administrative choices MS face when administering the benefit claims of Union citizens. The third section theorizes three typical administrative models for dealing with Union citizens’ access to social assistance. The fourth section presents the case studies on Germany, Austria and the Netherlands. In a subsequent fifth section, the discussion, we reflect on the different pathways of the three MS, and the factors that have influenced their choice of a specific administrative design, and place our analysis in a broader theoretical context. A concluding section summarizes and argues that the choice of a specific system has implications for the actual access of Union citizens to their ‘host’ welfare state and their experience of a ‘social Europe’ on the ground. While free movement law may have spurred the elimination of administrative restrictions on taking up residence in an increasing number of MS, we witness the emergence of institutionalized uncertainty in the welfare state in return.

## Open borders, lawful residence and equal access to welfare

Traditionally, countries have employed different control measures to regulate migration, such as registration requirements and residence permits (Brochmann, 1999). Although EU MS vary in their respective immigration control and policies, most will have made use of such measures to govern the (longer-term) presence of Union citizens in their territory (for example, Hammar, 1985: especially chapter 9). As for ‘regular’ immigrants, completing such ‘administrative formalities’ is often crucial for successfully claiming social benefits on equal terms with national citizens at a later stage (Morris, 2003). As this section will explain, the rights, limitations and conditions under EU law force MS into making two bureaucratic choices: first, in reconsidering the domestic division of tasks and cooperation between welfare and migration authorities and, second, in reconsidering the added value of traditional migration control measures like registration requirements for determining Union citizens’ *subsequent* access to the welfare state.

### Who verifies? Lawful residence and equal treatment

Stratification according to socio-economic status identified by scholars is largely based on two (overlapping) legal grounds for exclusion: exceptions to the principle of equal treatment and the conditions attached to lawful residence. Drawing a legal distinction is important as it has administrative consequences: which authority is competent to do what and when and under what limitations?

The possibilities for MS to derogate directly from the general principle of equal treatment are limited under EU law. Mostly due to the activist case law of the ECJ, the scope of the equal treatment principle has expanded considerably since the 1980s: more and more categories of mobile Union citizens have come to enjoy access to their ‘host’ welfare state. In 1986, the ECJ ruled that mobile workers engaged in ‘effective and genuine’ part-time economic activities generating an income below the minimum subsistence level could rely on social assistance to supplement their income (*Kempf*, C-139/85). Around the turn of the millennium, the ECJ used the newly established status of Union citizenship to award social benefits to ‘lawfully residing’ *inactive* Union citizens (*Martínez Sala*, C-85/96; *Grzelczyk*, C-184/99). Partly in response to this case law, the ‘old’ MS laid down some exceptions to the principle of equal treatment in Directive 2004/38 (‘Citizenship Directive’), just before the accession of Central and East European states. According to Article 24(2) of this Directive, MS are not obliged to grant social assistance to economically inactive Union citizens in their first 3 months of residence or longer when Union citizens continue to seek work.

While Article 24(2) therefore offers MS some room to exclude short-term inactive and job-seeking Union citizens, most of the stratification identified by scholars is the result of increased use of *residence conditions* as a ground for denying access to welfare. For a period of up to 3 months, a valid identity card suffices for lawful residence (Art 6 Dir 2004/38), but for longer periods MS may require Union citizens to be either economically active or to have sickness insurance and ‘sufficient resources not to become a burden on the social assistance system’ (Art 7 Dir 2004/38). MS authorities can therefore rely on residence conditions to declare Union citizens ‘illegal’ and thereby deny them ‘a right to have rights’ and access to welfare in the first place. It should be emphasized, however, that EU law provides safeguards to protect Union citizens from losing their right of residence. MS may not verify the legality of a Union citizen’s residence ‘systematically’ and can only do so in specific cases of ‘reasonable doubt’ as to whether a Union citizen still satisfies the conditions (Art 14 Dir 2004/38). While an actual claim to social assistance may be a reason to verify the right of residence, the (consequent) decision to terminate a Union citizen’s residence cannot be taken ‘automatically’. MS can only terminate residence when they take into account the individual circumstances of the Union citizen, and establish that the person forms an ‘*unreasonable* burden’ on the social assistance system (*Grzelczyk*, paras 42–43). Union citizens are relieved from meeting the residence conditions once they acquire a right to permanent residence after 5 years of legal and continuous residence in their ‘host’ MS (Art 16 Dir 2004/38). From that moment, they enjoy access to social assistance on equal terms as national citizens.

The battlefield over access to social benefits under EU law has shifted from the right to equal treatment to the right of residence over the past two decades (as predicted by O’Leary, 1999). It has mostly been on the basis of residence conditions that MS have stepped up their efforts to ‘quarantine’ Union citizens (Kramer et al., 2018) and they found endorsement from the ECJ in *Dano* (C-333/13), *Alimanovic* (C-67/14) and *Commission v. UK* (C-308/14). The right to social benefits – social assistance but also child benefits – could be made *conditional* on compliance with residence conditions, as ruled by the ECJ (O’Brien, 2017). As a result, residence conditions have taken centre-stage in the government and administration of European mobility, but not so much in relation to actual ‘presence’ as such, but rather in relation to the welfare state. In fact, MS could be completely uninterested in actually expelling Union citizens from the state territory, as this is considered largely futile in an open border Europe (Davies, 2016). It follows that the *assessment* of residence conditions should be distinguished from the actual *enforcement* of residence conditions from an administrative perspective. But if ‘residence conditions’ are (merely) instrumental for conditioning access to social benefits, which state authority does what with respect to the administration of Union citizens’ welfare claims?

Hence, MS have a first bureaucratic choice, namely whether welfare authorities become responsible for deciding if Union citizens reside ‘lawfully’, or whether migration authorities remain involved in such a decision.

### When to verify? The (ambiguous) role of administrative formalities

In addition to the question of *who* verifies the right of residence, another question is *when* the right of residence is verified. That is to say, whether MS authorities verify Union citizens’ compliance with residence conditions *prospectively* – at the moment of registration before their extended stay in the ‘host’ MS – or (also) *retrospectively* – at the moment when Union citizens actually apply for social assistance.

Case law of the ECJ dating back to the 1970s has been highly influential in limiting the role ‘administrative formalities’ should play in the exercise of free movement rights. According to the Court, Union citizens derive their free movement rights *directly* from the Treaty and can therefore exercise them *independently* of any administrative act by MS authorities (*Royer*, C-48/75). The objective behind this jurisprudence has been obvious, namely, to boost the substantive enjoyment of their free movement rights and break the formalistic and arbitrary power of MS’ bureaucracies by *constitutionalizing* free movement rights (see Schmidt, 2018). Largely codifying the *Royer*-line of jurisprudence, the Citizenship Directive now conveys a somewhat ambiguous message. MS may require Union citizens to register with the relevant authorities if they intend to stay longer than the initial 3 months and require them to provide proof that they satisfy the residence conditions (Art 8). Article 25, however, prohibits MS from making any administrative formality a ‘precondition’ for exercising free movement rights under the Treaty. Union citizens who fail to comply with requirements to register are only liable to sanctions, and do not lose any of the substantial rights – including their social rights – they derive directly from EU law (Art 8).

In sum, administrative formalities still exist under EU law, but the ECJ has essentially undermined their ‘worth and purpose’ (Davies, 2003). This legal ambiguity leads MS to face another bureaucratic choice, namely, to reconsider the added value of registration requirements and residence documents for Union citizens, especially now that they cannot be held against those citizens when claiming social benefits.

## Models of administering Union citizens in need: Rationalities and implications

It follows from the previous section that MS face choices when it comes to administering lawful residence and the right to social assistance of Union citizens in an open border Europe. This section introduces a typology of administrative models to deal with this puzzle, and discusses their rationale as well as their implications for Union citizens (Table 1). The three types, which are coined the *form*, *signal* and *delegation* models, allow for describing and comparing the administrative systems of MS and analysing their (non-)transformation. It is important to mention that ‘real world’ administrative systems hardly ever fully conform to one (single) ‘type’; they may combine elements of different models, turn out to be incoherent or arbitrary in practice, or reveal sub-national differences. The contours of the models were first observed on the basis of an explorative, inductive case study into MS’ welfare/migration administrations. These observations provided the inspiration to theorize the effects of administrative formalities, and the respective roles of welfare and migration authorities in different constellations, on Union citizens’ access to social assistance over time and space.

**Table 1.** Typology of three models of administering residence and related social assistance rights.

Model	Registration and documentation	Verification of residence right	Social assistance rights	Rationale	Implications
Form	Requirement to register and apply for residence document	Prospectively, by migration (or other) authority before extended stay	Linked to formal evidence of legal status	Weberian bureaucracy, division of competence	Social assistance rights depend on initial compliance with bureaucratic formalities
Signal	Abolished	Retrospectively, by migration authority after ‘signal’ from welfare authority	Depending on cooperation between welfare and migration authorities	‘Reversed’ Weberian bureaucracy, division of competence	Prospect of removal deters Union citizens; *de facto* stratification
Delegation	Abolished	Retrospectively, by *welfare authority* upon welfare application	Only granted upon passing ‘right to reside test’	Controlling welfare ‘at the gate’	Formalized stratification

### The form model

The first type of administration centres around the rationale of the ‘magical act of the form’.^1^ In a *form* model, correctly satisfying registration procedures and obtaining residence documents is essential for mobile Union citizens when claiming social assistance. This model resembles the governance of third country nationals’ access to welfare who can claim benefits according to their respective residence title. In the case of Union citizens, MS make use of the opportunities offered by EU law to introduce a registration certificate and verify the residence status of Union citizens *prospectively*. Within 3 months of arrival, Union citizens must typically register with the competent authorities (often migration authorities)^2^ and prove their compliance with the conditions of lawful residence under EU law. Subsequent interactions with welfare authorities are *linked* with the satisfactory fulfilment of these initial bureaucratic procedures: the decision of whether to grant access to welfare benefits is formally or ‘bureaucratically’ linked with Union citizens’ ability to demonstrate their legal status, often by requiring the provision of the ‘right’ residence document or the ‘correct’ residence status in a database. This procedure is at odds with EU (case) law in the sense that it ignores the ‘constitutionalized’ status of Union citizens, that is, that they enjoy their rights directly from the Treaties, independent of the fulfilment of administrative procedures.

The logic behind the *form* model can be understood from the perspective of Weberian bureaucratic rationality (Weber, 1978: 956 ff). The *form –* in its meaning as a written document or the fulfilment of the formal procedure – operates as a technique for ensuring predictability and impartiality and supports the coherent functioning of an administration that is characterized by a division of labour between different specialized offices. Since the ‘gatekeepers’ of social authorities rely on the formal documentation available to them to make their decision, Union citizens can know what to expect once successfully in the possession of the official paperwork. Bureaucratic reliance on the form can therefore work both to the advantage and disadvantage of Union citizens: some might gain access thanks to previously issued residence documents, to which they might not be substantively entitled at the moment of applying. Others, however, might be denied the possibility to claim their substantive rights under EU law because of difficulties they experience in assembling the required paperwork, or failures that occurred while navigating immigration procedures (see Brubaker, 1989: 160). It is at this point, when ‘faceless’ bureaucrats prioritize a commitment to the *form* over the *substantive* serving of the rights of citizens (Davies, 2003), that the positive aspects of Weberian bureaucratic rationality can slip into ‘moral distancing’ and arouse Kafkaesque feelings of alienation and dehumanization (Huber and Munro, 2013).

When effectively mobilized, EU law threatens to destabilize the coherent functioning of the *form* model (Greer and Rauscher, 2011). After all, it allows for the continued existence of residence documentation for Union citizens but prohibits attaching constitutive force to such documents in the realm of the welfare state. Sooner or later, this will trigger welfare administrations to stop regarding residence documentation as proof of welfare entitlement. In practice the result can be a confusing system for both welfare authorities’ caseworkers and Union citizens: welfare authorities cannot attach value to formal documentation certifying a successful passing of the *prospective* verification and Union citizens who do not register can still claim access to social assistance by directly relying on the substance of EU law. On the other side, Union citizens who duly fulfil procedures and obtain their documentation are given a false sense of security as they may perceive the document as title. Oddly enough, this situation can also be created by the implementation of the inherently ambiguous Citizenship Directive.

Over time, MS are therefore likely to abolish prospective verifications at the moment of registration but intensify retrospective verifications with respect to Union citizens who actually apply for social benefits, by requesting them to present evidence concerning their lawful residence for the period since their arrival. When leaving the *form* model behind, MS can move towards a *signal* model or a *delegation* model.

### The signal model

In the second model, social authorities are required to communicate with migration authorities when they grant social assistance to mobile Union citizens. It is the very claim to the welfare state that *informs* a subsequent decision by the migration authority about the right of residence of Union citizens. Due to the absence of initial residence verifications, the residence status of foreign Union citizens residing in the MS remains *unknown* in many cases and will only be ‘officially’ determined when they appear on the radar of migration authorities through ‘signals’ sent by the welfare authorities. The design and implementation of this model is challenging for a state administration as it requires a consistent and complex system of information exchange between welfare and migration authorities.

In a pure *signal* model, Union citizens are treated equally with respect to access to social benefits, but their official interaction with the welfare state can *potentially* have residential consequences. The metaphorical ‘gate’ towards social citizenship is initially open, but is characterized by a highly personalized *retrospective* construction of their conduct by the migration authority that might eventually result in a termination of residence and an order to leave the country. As Lafleur and Mescoli have pointed out, the welfare state acts as if ‘schizophrenic’: social authorities continue to fulfil their social function with respect to immigrants while migration authorities interpret such welfare use as a reason to exclude them (Lafleur and Mescoli, 2018: 493). This has important implications for actual access to welfare. As the very reason for applying for social assistance can invalidate their right of residence, the law applies forcefully in its openness and can deter Union citizens from engagement with the welfare system in the first place (Blauberger and Schmidt, 2014: 4; by analogy Agamben, 1998: 55). When MS do not actually expel Union citizens whose residence rights have been terminated, or when Union citizens strategically decide not to apply for benefits, the result of this model is one of *de facto* stratification of social rights: Union citizens factually reside in the MS’ territory but are excluded from social assistance and other benefits that require lawful residence.

### The delegation model

In the third model, MS have delegated the competence to assess residence conditions to welfare authorities when they decide on granting welfare benefits to Union citizens. Social authorities interested in welfare containment – for political or budgetary reasons – can now control ‘at the gate’ by integrating a so-called ‘right to reside test’ (O’Brien, 2017) into their general decision of whether to grant a benefit or not. In other words, they are not required to cooperate with migration authorities when excluding Union citizens based on the residence conditions under EU law. This *delegation* model rules out miscommunication between authorities, but implies a shift in the administrative burden and professional expertise on EU free movement law to welfare authorities.

As for the experience of Union citizens, they will have a ‘one stop shop’. But since migration authorities are no longer involved in decision-making, MS adopting a *delegation* model will typically no longer act in terms of explicit removal measures. Whereas the *signal* model still links welfare claims with lawful territorial presence, meaning that claiming benefits may result in a formal loss of the right to residence, the implications of applying for benefits in a *delegation* model remain limited to the welfare sphere. The *delegation* model hence implies a welfare state with an ‘accepted’ form of stratification: certain Union citizens are ‘tolerated’ on the territory of the MS but do not have access to social benefits (Nic Shuibhne, 2015). This form of stratification differs from the *de facto* stratification outlined above insofar as it is not created by a ‘simple’ lack of action, that is, by a lack of removal decision or the Union citizen’s fear of applying for social assistance, but it is the result of a systematic viewpoint that Union citizens can reside in the state territory but do not enjoy certain social rights.

## Empirical evidence

This section compares the (non-)transformation of three countries’ administration of social assistance benefits for persons capable of working, *Arbeitslosengeld II* in Germany, *Sozialhilfe* (until 2010) and *bedarfsorientierte Mindestsicherung* (since 2010) in Austria, and *sociale bijstand* in the Netherlands. The three MS all possess advanced welfare states that have experienced the arrival of Union citizens since the mid-2000s, yet they followed different trajectories of administrative change in terms of determining the eligibility to social assistance. Our analysis draws on government documents and working instructions signifying legislative and policy changes, as well as domestic case law. Moreover, we conducted 78 semi-structured interviews (19 in Germany, 35 in Austria, 24 in the Netherlands), mostly with welfare and migration authorities, within relevant ministries or central institutions responsible for social assistance, but also with rights advocacy groups or interest groups (for details see Supplemental Appendix 1). During these interviews, we asked – inter alia – about administrative practices and procedures, the reasons and motivations behind administrative decisions, possible changes over time and interactions with other state authorities.

### Germany (2004–2018): Completing its path to a delegation model

In Germany, three periods of administrative adjustment can be identified. From 2004 to 2013 (period 1), Germany functioned like a delegation model in practice, although residence documents certifying lawful residence were still in place (hence suggesting a *form* model). In 2004, the so-called free movement certificate (*Freizügigkeitsbescheinigung*) replaced the former residence document *Aufenthaltserlaubnis-EWG*. According to the Ministry of the Interior, the residence document for Union citizens had always been considered as declaratory, as prescribed by EU law, but had been preserved in order to help Union citizens in their daily life by proving their right of residence when renting flats or signing employment contracts (interview Ministry of the Interior, May 2017).

EU residence conditions form part of German social legislation. From 2007 to 2016, the respective law, Social Code II, stipulated that Union citizens whose right of residence solely arises out of their search for employment do not have access to social assistance, and that Union citizens – besides workers or self-employed persons – are excluded during the first 3 months of their stay. From 2016 onwards, §7 Social Code II also excludes Union citizens who do not have a right of residence. *Arbeitslosengeld II* is organized by a special authority, the Federal Employment Agency (*Bundesagentur für Arbeit*). Its head office compiles ‘special instructions’ in order to assist caseworkers in the local authorities, the so-called *Jobcenters* (interview Federal Employment Agency, April 2017). The special instructions on §7 Social Code II specify the conditions under which Union citizens have access to the benefit and highlight that the registration certificate is not decisive in proving lawful residence. Moreover, they lay down that economically ‘inactive’ Union citizens are excluded from the benefit as they do not have a right to reside.^3^

Having social legislation and administrative guidelines at their disposal, *Jobcenters* have thus, since 2004, typically assessed the residence conditions of Union citizens applying for benefits without involving the migration authority. Despite the existence of the registration certificate until 2013, *Jobcenters* considered whether Union citizens had a right of residence retrospectively, not taking the certificate into account (interviews *Jobcenters*, March, April 2016), and this practice was not questioned by domestic courts. In sum, we find a largely destabilized version of the *form* model early on: the registration certificate existed but did not guarantee welfare entitlement – a confusing situation for Union citizens.

While Germany already functioned like a *delegation* model in practice during the first period, it formally transformed its administrative system towards this model when the Ministry of the Interior abolished the registration certificate in 2013 (period 2).^4^ The Ministry received feedback from migration authorities that issuing the certificate was costly in terms of workload, and the usefulness of this certificate was limited since it was declaratory, characterized by a fast assessment and not tamper-proof. Moreover, Union citizens often perceived it (falsely) as a title (interview Ministry of the Interior, May 2017). The abolition of the registration certificate in 2013 had no consequences for most social authorities, as they did not consider it anyway and rejected the claims of non-active Union citizens on the basis of the law and the guidelines. For Union citizens, it was, however, a step towards legal clarity as far as the moment of assessment was concerned. With the registration certificate abolished, it became clear that lawful residence was only assessed *retrospectively*, at the moment of claiming benefits. At the same time, however, the abolition of the registration certificate also meant a step towards institutionalized uncertainty (arguably a product of ambivalent EU law) as regards the residence status of Union citizen, which is now uncertain and will only be determined after an application for social assistance. Germany thus completed its transition towards the *delegation* model. Welfare authorities control ‘at the gate’: they apply EU residence conditions and thus become delegated agents of migration policy. As a result, certain Union citizens are tolerated on German territory but denied access to social assistance.

After a legal reform in 2016, *Jobcenters* are now required to inform migration authorities about Union citizens who apply for benefits but are not considered to have the right of residence (interviews *Jobcenters* and migration authorities, March, April, June 2016, September 2017).^5^ Therefore, it is characteristic of this third period that economically inactive Union citizens who are denied social assistance by welfare authorities might face additional consequences regarding their right of residence (interview Ministry of the Interior, May 2017).^6^ Embracing elements of a *signal* model, this development is likely to have a deterrent effect on Union citizens, who may abstain from applying for benefits to avoid appearing on the radar of migration authorities.

### Austria (2006–2018): A fragmented system

Although Austria used to function as a pure *form* model in 2006, several federal districts have moved towards a delegation model in practice over time, resulting in a largely fragmented system. In 2006, Austria made use of the possibility offered by the Citizenship Directive to introduce a registration certificate (*Anmeldebescheinigung*). In order to obtain the certificate, Union citizens have to apply within the first 4 months of their arrival, and demonstrate that they meet the criteria for lawful residence.^7^ As part of its federal structure, Austrian social assistance falls within the competence of the nine regions. In contrast to Germany, there are nine different regional laws and there is no central Austrian-wide, special authority responsible for social assistance which could provide local welfare authorities with detailed guidelines.

In 2006, all regional laws linked access to social assistance to a right of residence. When Union citizens sought to claim benefits, the district administrations (*Magistrate* or *Bezirkshauptmannschaften*), the local welfare authorities, typically relied on the registration certificate as proof of their lawful residence (interviews social authorities, October, November 2015, January, October 2016). Whether Union citizens complied with the residence conditions under EU law was therefore only assessed *prospectively*, namely at the very start of their stay. Once issued with the certificate, the door to social assistance was basically open. In contrast, Union citizens who never obtained the certificate in the first place were denied benefits. The Austrian system of determining Union citizens’ right to social assistance clearly revolved around the ‘magical act of the form’ and therefore firmly fell within the *form* model.

Over time, due to specific concerns, more and more welfare authorities started to ask the migration authority to review the right of residence of Union citizens who claimed social assistance (interviews social and migration authorities, October, November 2015, January, October 2016). Once issued, however, the registration certificate was rarely revoked (interviews Ministry of the Interior, September 2016). Union citizens receiving social assistance ‘only’ had to live with the threat of being expelled. Although migration authorities stopped issuing certificates to economically inactive Union citizens over time (interviews migration authorities, October, November 2015, January 2016), the consequences of the separation of competences between migration and welfare authorities turned out to be more generous than intended (Heindlmaier and Blauberger, 2017).

Aware of the declaratory character of the registration certificate, and of the fact that it was rarely revoked by the migration authority, welfare authorities from the larger cities dealing with a large number of social assistance applications changed their practices by taking over the (original) role of the migration authority (Heindlmaier, 2020). After Union citizens applied for social assistance, officials would *retrospectively* assess their lawful residence themselves, without considering registration certificates or consulting the migration authority (interviews social authorities, January, October 2016). Vienna went so far as to modify its social legislation in 2010 for this purpose: only those Union citizens who were economically active or had a permanent right of residence would be entitled to social assistance.

In practice, the current situation is a highly fragmented administration. Some local welfare authorities, notably in rural areas, still treat the registration certificate as constitutive, while other authorities, often in urban areas, treat it as declaratory. From the perspective of Union citizens, the administrative apparatus must appear confusing, as a ‘tombola’ that awards social rights depending on the city or region where those rights are claimed. Two factors have arguably contributed to this metaphorical ‘tombola’: politics and courts. First, civil servants from the Ministry of the Interior are aware of the shortcomings of the registration certificate but cling on to it for political reasons. An important explanation seems to lie in public discourse. The Austrian government has always stressed the importance of the certificate as a means to exclude certain Union citizens from welfare benefits, given the link between certificate and social rights.^8^ Moreover, policymakers emphasize that the certificate assumes a big role in terms of ‘control’, that is, to know who resides in Austria, and for the purposes of deterrence (interview Ministry of the Interior, January 2016). The belief in the registration certificate thus still seems high enough to prevent Austria from abolishing it. Second, the national judiciary has not been able to create order in the ‘tombola’, since it has been receptive but ambivalent towards the jurisprudence of the ECJ. Whereas various domestic courts had earlier confirmed the declaratory character of the registration certificate,^9^ the Supreme Court of Justice opted for a constitutive interpretation of the registration certificate in its final ruling after the ECJ’s *Brey* judgement in 2013 (C-140/12): only the migration authorities were competent to assess the right of residence.^10^ This was, however, overturned after *Dano*: in May 2016, the Supreme Court ruled that the registration certificate only had a declaratory character and that Union citizens’ lawful residence may be reviewed at the moment they apply for benefits without involving the migration authority.^11^ This paved the way back towards a ‘tombola’ in Austrian administrative practice.

### The Netherlands (2001–2018): From form to signal

In the Dutch case, three periods of administrative adjustment can be identified. During the first period (2001–2007), Union citizens were required to apply for a residence document with the Immigration and Naturalization Service (‘Immigration Service’) after 6 months of residence. During an appointment they had to prove that they were either employed or possessed *independent* and *durable* sufficient resources for a period of at least a year, whereby ‘sufficient’ was defined as at least the social assistance norm.^12^ As for ‘regular immigrants’, Union citizens’ access to social assistance was governed through the ‘Linkage Act’, which ‘linked’ access to welfare with lawful residence (Noordam and Vonk, 2011: 54). When applying for social assistance at the municipalities, the local welfare authorities, Union citizens had to demonstrate their lawful residence by possessing a valid residence document and having the ‘correct’ residence status in the ‘Aliens Database’.^13^

This practice of ‘linking’ access to social assistance to the prior fulfilment of registration and documentation requirements came under increased scrutiny when a majority of national courts – including its highest social court, the Central Appeals Tribunal – scrutinized practices of municipalities that attached constitutive force to residence permits and residence ‘codes’ in the database. Instead, they ordered the municipalities to respect the independent status of Union citizens on the basis of EU law.^14^ Over the same period, the European Commission successfully challenged the strict registration requirements imposed by the Netherlands on economically inactive Union citizens before the ECJ (*Commission v. Netherlands*, C-398/06).

Due to these adjustment pressures, in combination with the implementation of the Citizenship Directive, the original *form* model became largely dysfunctional during the second period (2006–2013). From 2006 onwards, migration policy explicitly stated that the lawful residence of Union citizens did not depend on the possession of a residence document. After 4 months, however, Union citizens were still required to register with the Immigration Service in order to obtain a registration certificate (the ‘sticker’), whereby a ‘relatively flexible’ verification process would take place.^15^ Municipalities were required to grant social assistance to Union citizens and subsequently notify the Immigration Service, which would have to decide on their right of residence. However, since the Immigration Service did not process these notifications due to ‘legal complexity’ and ‘other policy priorities’,^16^ municipalities were incentivized to act restrictively on their own accord in a process reminiscent of the Austrian case. Some would refuse social assistance to Union citizens who had failed to register by treating them as ‘workseekers’, others would independently refuse social assistance on the basis of the residence conditions formulated in the Citizenship Directive (interviews municipalities, January, February and April 2016). At least until 2013, Dutch administration of Union citizens’ access to social assistance was largely uncoordinated, confusing and arbitrary in practice, a ‘system’ described by one official as a ‘tombola’ (interview municipality April 2016).

While politicians had already stressed the need to establish effective cooperation between municipalities and the migration authority,^17^ the final reforms towards a *signal* model (2013–present) were eventually triggered by a crucial judgement of the Central Appeals Tribunal in 2013. The Tribunal firmly declared that the municipalities were not authorized to reject social benefits to Union citizens on the basis of residence conditions.^18^ Instead, municipalities had to grant social assistance to Union citizens residing longer than 3 months for as long as the Immigration Service had not taken an explicit decision on their residence status. Clearly dividing competences, the judgement placed the workload of assessing residence conditions on the Immigration Service (interviews Ministry of Security and Justice, January 2016, and Immigration Service, February 2016). After applying for social assistance, Union citizens can now expect a letter from the Immigration Service containing a detailed set of questions about their activities since their arrival in the Netherlands. Based on the evidence presented by Union citizens, case workers in the Immigration Service ‘reconstruct’ their situation and activities under EU law and decide whether or not to terminate their right of residence. If residence is terminated, the municipalities will stop granting social assistance and the Union citizens will receive a letter demanding that they leave the country within 4 weeks or otherwise face removal. In 2014, the Netherlands also abolished the requirement for Union citizens to register, for similar reasons as Germany. Instead of providing Union citizens with a ‘false sense of certainty’ at the moment of registration, as the authorities explained their decision, the procedure of testing residence conditions would now be ‘information led’.^19^

The combined result of these reforms is the establishment of a system that could be described as a strong case of a *signal* model. In contrast to ‘regular aliens’, the residence status of Union citizens residing in the Netherlands remains *unknown* in many cases and will only be determined when they appear on the radar of the Immigration Service through the bureaucratic procedures of the welfare state. For Union citizens who are not likely to meet the residence conditions, this system might turn out to be generous, as they get to receive social assistance until the consequences for their residence are processed. For others, however, the threat of losing the right of residence hangs over their head like the sword of Damocles: while for some the order to leave the country will come as an unpleasant surprise, those Union citizens aware of the possible consequences might opt to live a life under the minimum subsistence level (Kramer, 2017).

## Discussion

Our comparative case studies relating to Germany, Austria and the Netherlands demonstrate how MS have responded differently to the complex puzzle of dealing with open borders, the administration of lawful residence and the right to social assistance over time. Furthermore, they also shed light on the motivations and domestic factors that influenced different pathways of administrative change.

Both Germany and the Netherlands made the strategic decision to stop monitoring Union citizens’ compliance with free movement law ‘at entry’ and to abolish corresponding residence documentation. Their motivation was to decrease the administrative burden for migration authorities and limit the (unintended) consequences of issuing a ‘worthless’ residence document, which merely caused confusion in the wider (welfare) state administration and for Union citizens themselves, who were given a false sense of (social) security. Through a process of learning, defined as ‘judgments about whether [. . .] a given policy tool is still preferred relative to alternatives’ (May, 1992: 333), Germany and the Netherlands came to experience residence documents as policy instruments of ‘error’. Their preferred alternative, one that would both serve the political objective of restricting welfare provision to certain Union citizens and would be ‘ECJ-proof’ (Blauberger, 2012), was to abolish registration requirements and organize administrative procedures that *retrospectively* verify lawful residence of only those Union citizens who actually claim social assistance.

While the law might ultimately encourage all MS to move away from the *form* model in a similar fashion, such a decision cannot be taken for granted in every single MS. As our case studies suggest, two factors in particular can delay or even prevent administrative adjustment. A first factor concerns a strongly held, politicized belief (May, 1992) in the merit and rationale behind registrations and residence documentation as effective policy instruments. As the case of Austria illustrates, decision makers may cling to such formalistic procedures when they believe these to be effective instruments to maintain ‘control’ over who resides in the country (Wright, 2016) while neglecting the implications this can have on the ground. Whereas isolated action by regional authorities that face a high number of (similar) welfare requests, and judicial responses to European case law, currently appear to trigger adjustment towards a *delegation* model, a lack of coordinated reform has so far left Austria with a confusing and fragmented bureaucratic system in practice.

Another factor concerns uneven patterns of mobilization and enforcement of EU law before national courts (Conant et al., 2017). Particularly in a situation when the European Commission does not take direct action, national judges play a crucial role in generating adjustment pressures and forcing MS authorities to reconsider their policy instruments (Conant, 2002: 200–207). Compared to relatively active courts in the Netherlands and Germany, for example, those in the ‘Nordic’ MS have proven rather reluctant to push for Europe (Kramer et al., 2018: 1506; Wind, 2010). This might explain why, for example, Sweden seems to get away with ignoring the ‘constitutionalized’ status of Union citizenship by sticking to a compulsory registration system, including a *prospective* ‘right to reside’ assessment. Becoming a formalized resident in Sweden and receiving a personal ID number (*personnummer*) is not easy, as the Swedish Tax Agency prospectively verifies ‘a person’s capability to continuously fulfil the Directive’s requirements for lawful residence for at least one year’ (Hyltén-Cavallius, 2018: 139). While obtaining the *personnummer* can therefore be notoriously difficult, possessing it after a successful registration serves as the basic proof of the individual’s inclusion in the largely residence-based Swedish welfare state (Bruzelius, 2019; Erhag, 2016). Hence, Sweden appears to be the clearest example of a real-world *form* model.

While Germany and the Netherlands both decided to abolish registration requirements ‘at entry’, they arrived at different administrative systems to legally determine Union citizens’ residence status. The explanation for this variation seems to lie in the particular domestic relationship between migration and social legislation, as well as the functional division of competences between administrative authorities. Germany was able to ‘delegate’ the verification of residence conditions to local welfare authorities by incorporating those conditions into social legislation and administrative guidelines, allowing those authorities to assess the right to social assistance ‘at the gate’. The German administrative system therefore fits the *delegation* model. A similar claim can be made with respect to the United Kingdom, which has directly incorporated a range of ‘right to reside’ tests into its social legislation over the past 15 years (Barbulescu and Favell, 2020: 155–158). While the Dutch administration has also tried to move towards a *delegation* model, domestic courts have been adamant in maintaining a clear division of competences between the municipalities as the providers of social assistance (on the basis of social legislation), and the Immigration Service as the executive of migration law.^20^ The outcome is typical for a *signal* model: if welfare authorities want to restrict welfare access on the basis of EU residence conditions, they will have to ‘signal’ to, and cooperate with, the migration authority.

## Conclusion

The complex combination of having open borders and mobile Union citizens with a ‘constitutional’ status but a conditional right to residence determining equal access to social assistance was bound to shake up established procedures and divisions of competences between welfare and migration authorities in EU MS. While concerted in their move towards restrictive policies against a background of increased politicization, Western European MS respond(ed) differently to the puzzle of exactly how to administer Union citizens in need. In this contribution, we developed a typology of how MS can respond to the puzzle: the *form*, *signal* and *delegation* models. By analytically distinguishing between these three administrative models, we were able to trace the adjustments that were made in Germany, Austria and the Netherlands with respect to the administrative structures and procedures dealing with Union citizens who have recourse to their social assistance systems. While our analysis was limited to social assistance in the three MS, this typology could be extended to other MS and, in fact, any other non-contributory benefit that MS link to the right of residence under EU law, a legacy left by the ECJ in its (vain) attempt to keep the United Kingdom within the EU (O’Brien, 2017).

Differences between bureaucratic systems – as adapted to the Union’s free movement regime – matter for both the experience of Union citizens of their social rights, and their actual access to the social benefits of the ‘host’ state. Whereas an early attachment to the ‘form’ might have excluded Union citizens on purely formal grounds or created uncertainty about their actual residence status, the current trend towards abolishing residence documentation entails a *de facto* institutionalization of legal uncertainty: the residence status of many Union citizens is simply *unknown*. A retrospective verification of compliance with EU law – and the possible prospect of illegality – not only shifts the burden of proof to the individual Union citizens, but can also deter them from engaging with the ‘host’ welfare state in the first place. Although, compared to third-country nationals, Union citizens could still be considered ‘privileged’ immigrants in many ways, the ‘formless’ character of their free movement contributes to their social precarity. The stratification of social rights, in other words, is not only the result of legal and political decision-making, but also of bureaucratic design (similarly, Bruzelius, 2019). The alternative ways in which MS bureaucracies administer mobile Union citizens therefore also offer important insights into the functioning of territorial welfare states in open border regimes.

## Supplemental Material

sj-pdf-1-esp-10.1177_0958928721999612 – Supplemental material for Administering the Union citizen in need: Between welfare state bureaucracy and migration control Click here for additional data file. Supplemental material, sj-pdf-1-esp-10.1177_0958928721999612 for Administering the Union citizen in need: Between welfare state bureaucracy and migration control by Dion Kramer and Anita Heindlmaier in Journal of European Social Policy

## Notes

1. We thank Clemens Kaupa for suggesting this lovely phrase.

2. In Sweden, for example, tax authorities are the competent authorities (see Discussion).

3. For instance: special instruction from 20 December 2011.

4. Bundestag, Drucksache 17/10746.

5. Special instruction from 20 January 2016, point 7.53a.

6. Bundestag, Drucksache 18/1602.

7. §53 and §51 Residence and Settlement Act.

8. For instance: ‘Barrieren gegen den Missbrauch’, *Kleine Zeitung*, 8 January 2014.

9. For instance: Verwaltungsgerichtshof, 30-01-2007, 2006/21/0330.

10. Oberster Gerichtshof der Republik Österreich, 17-12-2013, 10ObS152/13w.

11. Oberster Gerichtshof der Republik Österreich, 10-5-2016, 10ObS 15/16b.

12. *Vreemdelingencirculaire 2000*, B10/4.2.1.1 (version of 1 April 2001).

13. *Vreemdelingencirculaire 2000*, B10/2.6 (version of 1 April 2001). Domestic case law demonstrates this practice. Rechtbank Arnhem, 17-09-2004, ECLI:NL:RBARN:2004:AR3129, Rechtbank Maastricht, 23-12-2005, ECLI:NL:RBMAA:2005:AU9591, Recht-bank Maastricht, 11-04-2006, ECLI:NL:RBMAA:2006:AX3093 and Rechtbank Middelburg, 23-03-2007, ECLI:NL:RBMID:2007:BB5134. Also, Om-budsman Amsterdam Zuid-Oost, ‘Gemeentelijke Basisadministratie, Afwijzing bijstandsaanvraag; Sociale Dienst’, 11-05-2005.

14. Centrale Raad van Beroep, 13-06-2006, ECLI:NL:CRVB:2006:AY3868.

15. *Vreemdelingenbesluit 2000*, Art 8.12 (4). *Staatsblad* 2006, no. 215, p. 22.

16. As follows from an internal report, Inspectie Werk en Inkomen/Informatie-en Analysecentrum IND, ‘Bijstand, WW en verblijfsvergunning’, 1-12-2008.

17. Parliamentary document 2008–2009, 29,407, no. 100, pp. 3, 4 and 24. Parliamentary document 2007–2008, 29,407, no. 88.

18. Centrale Raad van Beroep, 19-03-2013, NL:CRVB:2013:BZ3857.

19. *Staatsblad* 2014, 268, 8 July 2014.

20. Centrale Raad van Beroep, 22-5-2018, ECLI:NL:CRVB:2018:1750.

## References

[bibr1-0958928721999612] Agamben G. (1998) Homo Sacer: Sovereign Power and Bare Life. Stanford, CA: Stanford University Press.

[bibr2-0958928721999612] Barbulescu R. Favell A. (2020) ‘Commentary: A Citizenship without Social Rights? EU Freedom of Movement and Changing Access to Welfare Rights’, International Migration 58: 151–165.

[bibr3-0958928721999612] Blauberger M. (2012) ‘With Luxembourg in Mind. . . the Remaking of National Policies in the Face of ECJ Jurisprudence’, Journal of European Public Policy 19: 109–126.

[bibr4-0958928721999612] Blauberger M. Schmidt S.K. (2014) ‘Welfare Migration? Free Movement of EU Citizens and Access to Social Benefits’, Research and Politics 1: 1–7.

[bibr5-0958928721999612] Blauberger M. Schmidt S.K. (2017) ‘Free Movement, the Welfare State, and the European Union’s Over-Constitutionalization: Administrating Contradictions’, Public Administration 95: 437–449.

[bibr6-0958928721999612] Blauberger M. Heindlmaier A. Kramer D. Martinsen D. Sampson Thierry J. Schenk A. , et al. (2018) ‘ECJ Judges Read the Morning Papers: Explaining the Turnaround of European Citizenship Jurisprudence’, Journal of European Public Policy 25: 1422–1441.

[bibr7-0958928721999612] Brochmann G. (1999) ‘The Mechanisms of Control’, in Brochmann G. Hammar T. (eds.) Mechanisms of Immigration Control: A Comparative Analysis of European Regulation Policies, pp.1–27. Oxford: Berg.

[bibr8-0958928721999612] Brubaker R. (1989) ‘Membership Without Citizenship: The Economic and Social Rights of Noncitizens’, in Brubaker R. (ed.) Immigration and the Politics of Citizenship in Europe and North America, pp.145–162. Lanham, MD: University Press of America.

[bibr9-0958928721999612] Bruzelius C. (2019) ‘Freedom of Movement, Social Rights and Residence-Based Conditionality in the European Union’, Journal of European Social Policy 29: 70–83.

[bibr10-0958928721999612] Bruzelius C. Seeleib-Kaiser M. (2017) ‘European Citizenship and Social Rights’, in Kennett P. Lendvai-Bainton N. (eds.) Handbook of European Social Policy, pp.155–166. Cheltenham, UK: Edward Elgar.

[bibr11-0958928721999612] Carmel E. Paul R. (2013) ‘Complex Stratification: Understanding European Union Governance of Migrant Rights’, Regions and Cohesion 3: 56–85.

[bibr12-0958928721999612] Conant L. (2002) Justice Contained: Law and Politics in the European Union. Ithaca, NY: Cornell University Press.

[bibr13-0958928721999612] Conant L. Hofmann A. Soennecken D. Vanhala L. (2017) ‘Mobilizing European Law’, Journal of European Public Policy 25: 1376–1389.

[bibr14-0958928721999612] Davies G. (2003) ‘Bureaucracy and Free Movement: A Conflict of Form and Substance’, Nederlands tijdschrift voor Europees recht 4: 81–89.

[bibr15-0958928721999612] Davies G. (2016) Migrant Union Citizens and Social Assistance: Trying to be Reasonable About Self-Sufficiency. Research Paper in Law 2/2016. Bruges, Belgium: College of Europe/European Legal Studies.

[bibr16-0958928721999612] Erhag T. (2016) ‘Under Pressure? Swedish Residence-Based Social Security and EU Citizenship’, European Journal of Social Security 18: 207–231.

[bibr17-0958928721999612] Ferrera M. (2014) ‘Social Europe and its Components in the Midst of the Crisis: A Conclusion’, West European Politics 37: 825–843.

[bibr18-0958928721999612] Greer S. Rauscher S. (2011) ‘Destabilization Rights and Restabilization Politics: Policy and Political Reactions to European Union Healthcare Services Law’, Journal of European Public Policy 18: 220–240.

[bibr19-0958928721999612] Hammar T. (1985) European Immigration Policy: A Comparative Study, Cambridge: Cambridge University Press.

[bibr20-0958928721999612] Heindlmaier A. (2020) “Social Citizenship” at the Street Level? EU Member State Administrations Setting a Firewall’, Journal of Common Market Studies.Epub ahead of print 30 January 2020. DOI: 10.1111/jcms.13028. PMC7508057 32999507

[bibr21-0958928721999612] Heindlmaier A. Blauberger M. (2017) ‘Enter at Your Own Risk: Free Movement of EU Citizens in Practice’, West European Politics 40: 1198–1217.

[bibr22-0958928721999612] Huber C. Munro I. (2013) “Moral Distance” in Organizations: An Inquiry into Ethical Violence in the Works of Kafka’, Journal of Business Ethics 124: 1–11.

[bibr23-0958928721999612] Hyltén-Cavallius K. (2018) ‘The Need of Residence Registration for Enjoyment of EU Citizenship in Sweden’, in Pennings F. Seeleib-Kaiser M. (eds.) EU Citizenship and Social Rights: Entitlements and Impediments to Accessing Welfare, pp.127–148. Cheltenham, UK: Edward Elgar.

[bibr24-0958928721999612] Kramer D. (2017) ‘Kostendelersnorm, bijstand en Unieburger: Koppelingswet 2.0?’, Asiel & Migrant-enrecht 2017: 4–9.

[bibr25-0958928721999612] Kramer D. Sampson Thierry J. van Hooren F. (2018) ‘Responding to Free Movement: Quarantining Mobile Union Citizens in European Welfare States’, Journal of European Public Policy 25: 1501–1521.

[bibr26-0958928721999612] Lafleur J.-M. Mescoli E. (2018) ‘Creating Undocumented EU Migrants through Welfare: A Conceptualization of Undeserving and Precarious Citizenship’, Sociology 52: 480–496. 29899582 10.1177/0038038518764615 PMC5985588

[bibr27-0958928721999612] Mantu S. Minderhoud P. (2019) ‘Exploring the Links between Residence and Social Rights for Economically Inactive EU Citizens’, European Journal of Migration and Law 21: 313–337.

[bibr28-0958928721999612] May P. J. (1992) ‘Policy Learning and Failure’, Journal of Public Policy 12: 331–354.

[bibr29-0958928721999612] Morris L. (2003) ‘Managing Contradiction: Civic Stratification and Migrants’ Rights’, The International Migration Review 37: 74–100.

[bibr30-0958928721999612] Nic Shuibhne N . (2015) ‘Limits Rising, Duties Ascending: The Changing Legal Shape of Union Citizenship’, Common Market Law Review 52: 889–938.

[bibr31-0958928721999612] Noordam F. Vonk G. (2011) Hoofdzaken sociale zekerheidsrecht. Deventer, Netherlands: Kluwer.

[bibr32-0958928721999612] O’Brien C. (2016) ‘Civis Capitalist Sum: Class as the New Guiding Principle of EU free Movement Rights’, Common Market Law Review 53: 937–978.

[bibr33-0958928721999612] O’Brien C. (2017) ‘The ECJ Sacrifices EU Citizenship in Vain: Commission v. United Kingdom’, Common Market Law Review 54: 209–244.

[bibr34-0958928721999612] O’Leary C. (1999) ‘Putting Flesh on the Bones of European Union Citizenship’, European Law Review 24: 68–79.

[bibr35-0958928721999612] Roos C. (2016) ‘Freedom of Movement in the EU and Welfare State Closure: Welfare Regime Type, Benefit Restrictions and Their Implications for Social Mobility’, in Wulfgramm M. Bieber T. Leibfried S. (eds.) Welfare State Transformations and Inequality in OECD Countries. Transformations of the State, pp.267–289. London: Palgrave Macmillan.

[bibr36-0958928721999612] Sainsbury D. (2012) Welfare States and Immigrant Rights: The Politics of Inclusion and Exclusion. Oxford: Oxford University Press.

[bibr37-0958928721999612] Schmidt S. K. (2018) The European Court of Justice and the Policy Process: The Shadow of Case Law. Oxford: Oxford University Press.

[bibr38-0958928721999612] Shutes I. (2016) ‘Work-related Conditionality and the Access to Social Benefits of National Citizens, EU and Non-EU Citizens’, Journal of Social Policy 45: 691–707.

[bibr39-0958928721999612] Soysal Y. (1994) Limits of Citizenship: Migrants and Postnational Membership in Europe. Chicago, IL: University of Chicago Press.

[bibr40-0958928721999612] Weber M. (1978) Economy and Society. Berkeley, CA: University of California Press.

[bibr41-0958928721999612] Wind M. (2010) ‘The Nordics, the EU and the Reluctance Towards Supranational Judicial Review’, Journal of Common Market Studies 48: 1039–1063.

[bibr42-0958928721999612] Wollenschläger F. (2011) ‘A New Fundamental Freedom beyond Market Integration: Union Citizenship and its Dynamics for Shifting the Economic Paradigm of European Integration’, European Law Journal 17: 1–34.

[bibr43-0958928721999612] Wright C. F. (2016) ‘Control Signals and the Social Policy Dimensions of Immigration Reform’, in Freeman G. P. Mirilovic N. (eds.) Handbook on Migration and Social Policy, pp.161–179. Cheltenham, UK: Edward Elgar.

