# Supplementary material for: Administering the Union citizen in need: Between welfare state bureaucracy and migration control
Source: J Eur Soc Policy. 2021 Apr 12;31(4):380–94. doi: 10.1177/0958928721999612 (PMC8521353; doi:10.1177/0958928721999612)
Supplement: sj-pdf-1-esp-10.1177_0958928721999612 – Supplemental material for Administering the Union citizen in need: Between welfare state bureaucracy and migration control [file sj-pdf-1-esp-10.1177_0958928721999612.pdf]

Appendix 1: Interviews conducted in Germany, Austria and the Netherlands

Country	Organisation	Date
Germany	Jobcenter 1	2016, March
	Jobcenter 2	2016, April
	Jobcenter 3	2016, April
	Jobcenter 4	2016, April
	Jobcenter 5	2016, April
	Jobcenter 6	2016, April
	Federal Employment Agency, head office	2017, April
	Federal Employment Agency, regional office	2017, April
	Rights advocacy group 1	2016, March
	Rights advocacy group 2	2016, April
	Rights advocacy group 3	2016, April
	Rights advocacy group 4	2016, April
	German County Association	2016, April
	Social unit of district administration 1	2016, April
	Social unit of district administration 2	2016, April
	Immigration unit of district administration 1	2016, April
	Immigration unit of district administration 2	2016, June
	Immigration unit of district administration 3	2017, September
	Ministry of the Interior	2017, May
Austria	Social unit of district administration 1	2015, October
	Social unit of district administration 2	2015, October
	Social unit of district administration 3	2015, October
	Social unit of district administration 4	2015, October
	Social unit of district administration 5	2015, October
	Social unit of district administration 6	2015, October
	Social unit of district administration 7	2015, October
	Social unit of district administration 8	2015, October
	Social unit of district administration 9	2015, October
	Social unit of district administration 10	2015, October
	Social unit of district administration 11	2015, October
	Social unit of district administration 12	2015, October
	Social unit of district administration 13	2015, October
	Social unit of district administration 14	2015, October
	Social unit of district administration 15	2015, November
	Social unit of district administration 16	2016, January
	Social unit of district administration 17	2016, October
	Social unit of provincial government 1	2015, December
	Social unit of provincial government 2	2016, January
	Ministry of Labour, Social Affairs and Consumer Protection 1	2016, January
	Ministry of Labour, Social Affairs and Consumer Protection 2	2016, September
	Immigration unit of district administration 1	2015, October
	Immigration unit of district administration 2	2015, October

	Immigration unit of district administration 3	2015, October
	Immigration unit of district administration 4	2015, October
	Immigration unit of district administration 5	2015, November
	Immigration unit of district administration 6	2015, November
	Immigration unit of district administration 7	2015, November
	Immigration unit of district administration 8	2015, November
	Immigration unit of district administration 9	2016, January
	Interest group	2016, January
	Ombudsman institution	2016, January
	Rights advocacy group	2016, September
	Ministry of the Interior	2016, January
	Ministry of the Interior, Federal Office for Immigration and Asylum	2016, September
Netherlands	Ministry of Social Affairs and Employment	2015, April
	Municipality 1	2016, January
	Municipality 1	2016, January
	Ministry of Social Affairs and Employment	2016, January
	Municipality 1	2016, January
	Municipality 2	2016, January
	Municipality 3	2016, January
	Municipality 3	2016, January
	Parliament	2016, January
	Ministry of Security and Justice	2016, January
	Municipality 4	2016, February
	Parliament	2016, February
	Immigration and Naturalisation Service	2016, February
	Immigration and Naturalisation Service	2016, February
	Immigration and Naturalisation Service	2016, February
	Municipality 5	2016, February
	Social welfare organisation	2016, March
	Municipality 5	2016, April
	Municipality 5	2016, April
	Ministry of Foreign Affairs	2016, June
	Ministry of Foreign Affairs	2016, June
	Rights advocacy group	2016, July
	Immigration and Naturalisation Service	2017, March
	Immigration and Naturalisation Service	2019, June
